# A Case of Recurrent Acute Pancreatitis Secondary to Hypertriglyceridemia

**DOI:** 10.7759/cureus.24223

**Published:** 2022-04-17

**Authors:** Kinza Iqbal, Sawai Singh Rathore, Nitesh K Jain, Simranjit Singh, Muthumeena Kannappan, Ramesh Adhikari

**Affiliations:** 1 Internal Medicine, Dow University of Health Sciences, Karachi, PAK; 2 Internal Medicine, Dr. Sampurnanand Medical College, Jodhpur, IND; 3 Critical Care Medicine, Mayo Clinic Health System, Mankato, USA; 4 Internal Medicine, Indiana University School of Medicine, Indianapolis, USA; 5 Hospital Medicine/Primary Care and Public Health, Franciscan Health, Lafayette, USA; 6 Hospital Medicine, Franciscan Health, Lafayette, USA; 7 Geriatrics, Brown University, Providence, USA

**Keywords:** hypertriglyceridemia, hypetrygliceridemic pancreatitis, hypertriglyceridemia induced pancreatitis, recurrent, acute pancreatitis

## Abstract

Hypertriglyceridemia is known to be the third most common etiology of acute pancreatitis. Triglyceride levels above 1,000 mg/dL are associated with an increased risk of acute pancreatitis. We present the case of a 22-year-old female, a known case of hypertriglyceridemia, who developed sudden onset severe epigastric abdominal pain. A marked elevation in triglyceride levels of >3,000 mg/dL, serum lipase levels of 722 U/L, and serum amylase levels of 161 U/L, in the absence of other risk factors of acute pancreatitis, suggested hypertriglyceridemia-induced acute pancreatitis. Computed tomography (CT) of the abdomen and pelvis with contrast confirmed acute pancreatitis with hepatic steatosis. She was initially placed nil per os (NPO) and intravenous (IV) fluids with normal saline were administered. However, she was subsequently transferred to the intensive care unit as she developed acute respiratory distress syndrome. She was started on IV insulin with 5% dextrose in normal saline and a hydromorphone hydrochloride patient-controlled analgesia (PCA) pump was used for pain control. The patient’s condition improved gradually. At the time of discharge, the triglyceride (311 mg/dL) and lipase levels (81 U/L) of the patient were within the normal range. The prognosis of hypertriglyceridemia-induced acute pancreatitis is considered to be worse than non-hypertriglyceridemic acute pancreatitis. Patients with hypertriglyceridemia-induced acute pancreatitis need swift diagnosis and treatment to avoid serious complications.

## Introduction

Acute pancreatitis is a common gastrointestinal cause of hospitalization in the United States [1]. It involves the sudden onset of inflammation of the pancreas, which presents with symptoms, including abdominal pain radiating to the back, nausea, vomiting, and/or fever [2]. While acute pancreatitis has many causative factors, hypertriglyceridemia is the third most common etiology after gallstones and alcohol abuse. Hypertriglyceridemia is defined as serum triglyceride concentration above 150 mg/dL; triglyceride levels over 1,000 mg/dL are classically regarded as a risk factor for acute pancreatitis [1]. Around 5% of the patients with serum triglyceride levels above 1,000 mg/dL develop acute pancreatitis, while the incidence of acute pancreatitis is much higher (10%-20%) with levels > 2,000 mg/dL [3]. Hypertriglyceridemia-induced acute pancreatitis often results in a severe disease course and increases the risk of life-threatening complications compared with other causes of acute pancreatitis [4]. Therefore, a prompt diagnosis and an appropriate treatment are crucial. The current paper presents a patient with recurrent acute pancreatitis due to markedly elevated serum triglyceride levels. The mechanisms implicated in hypertriglyceridemia-induced acute pancreatitis and the appropriate management are highlighted.

## Case presentation

A 22-year-old female with a history of hypertriglyceridemia, splenic vein thrombosis, diabetes mellitus type 2, class I obesity, history of severe familial hypertriglyceridemia presented to the emergency department with the chief complaint of a sudden episode of abdominal pain- severe epigastric pain. Abdominal pain started on the night of admission. The epigastric pain was sharp and stabbing in character and radiated to her back and left flank. It was exacerbated by movement and improved with pain medication. The patient also complained of nausea, chills, and diarrhea. She took Imodium (Loperamide) for diarrhea, which was effective to some extent.

The patient took fenofibrate 145 mg daily for hypertriglyceridemia (lowest levels - 293 mg/dL - seven months before admission) and metformin for diabetes mellitus (HbA1C-8.3%). The patient denied missing her medications. She had the last episode of acute pancreatitis seven months ago. She had recently completed a three-month course of anticoagulation with apixaban (Eliquis) for splenic vein thrombosis. She denied smoking, alcohol consumption, or drug abuse. The patient’s mother also had a history of hypertriglyceridemia with pancreatitis. However, she had no history of gallstones, procedures (including endoscopic retrograde cholangiopancreatography (ERCP) or appendectomy), or new medications.

Initial labs showed a normal metabolic panel with an elevated glucose level of 264 mg/dL (74-99 mg/dL). Liver function tests were normal, except for a mild elevation of Aspartate aminotransferase (AST), i.e., 30 U/L (13-39 U/L). A complete blood picture showed a mild elevation in the white blood cell count, i.e., 11.8 ×10^3^/µL (4.0-11.0 ×10^3^/µL), and hemoglobin levels, i.e., 15.8 g/dL (12.0-15.3 g/dL) most likely from hemoconcentration. Lactate dehydrogenase (LDH) levels were elevated, i.e., 400 U/L (140-271 U/L) on the day of presentation. Initial procalcitonin levels were normal. The fasting lipid panel showed triglycerides >3,000 mg/dL, total cholesterol 578 mg/dL, low density lipoprotein (LDL) 175 mg/dL, high-density lipoprotein (HDL) 12 mg/dL, and non-HDL 566 mg/dL.

Table 1 presents the initial laboratory values of the patient at the time of admission.

**Table 1:** Laboratory values CBC: complete blood count; WBC: white blood cells; RBC: red blood cells; MCV: mean corpuscular volume; MCH: mean corpuscular hemoglobin; MCHC: mean corpuscular hemoglobin concentration; RDW: red cell distribution width; MPV: mean platelet volume; SGOT: serum glutamic-oxaloacetic transaminase; A/G ratio: albumin to globulin ratio; HDL: high-density lipoprotein; VLDL: very-low-density lipoprotein; CHOL HDL-C ratio: total cholesterol to high-density lipoprotein cholesterol ratio; LDL: low-density lipoprotein; mg/dL: milligrams per deciliter; U/L: units per liter; g/dL: grams per deciliter; pg: picograms; fL: femtoliter; mmol/L: millimoles per liter; mEq/L: milliequivalents per liter; μL: microliter.

Laboratory parameters	Patient’s values	Normal values
CBC		
WBC (10*3/μL)	11.8	4.0 – 11.0
RBC (10*3/μL)	5.13	3.63 – 5.04
Hemoglobin (g/dL)	15.8	12.0 – 15.3
Hematocrit (%)	38.2	34.7 – 45.1
MCV (fL)	74.5	80.0 – 100.0
MCH (pg)	30.8	26.0 – 34.0
MCHC (g/dL)	41.4	32.5 – 35.8
RDW (%)	23.3	11.9 – 15.9
Platelets (103/μL)	332	150 – 450
MPV (fL)	8.0	6.8 – 10.2
WBC Differential		
Neutrophil %	71.5	43.0 – 82.3
Band Neutrophil %	0	0.0 – 10
Lymphocyte %	22.7	14.5 – 45.2
Monocyte %	4.5	4.3 – 13.3
Eosinophil %	0.7	0.1 – 6.8
Basophil %	0.6	0.0 – 2.0
CHEMISTRIES		
Sodium (mmol/L)	132	133 – 144
Potassium (mmol/L)	4.2	3.5 – 5.2
Chloride (mmol/L)	101	98 – 107
Carbon dioxide (mmol/L)	22	21 – 31
Anion gap (meq/L)	9	6.2 – 14.7
Blood urea nitrogen (mg/dL)	10	7 – 25
Creatinine (mg/dL)	0.7	0.6 – 1.2
Calcium (mg/dL)	8.9	8.6 – 10.3
Glucose (mg/dL)	264	70 – 99
Total Alkaline Phosphatase (U/L)	57	34 – 104
Total protein (g/dL)	7.9	6.4 – 8.9
Albumin (g/dL)	4.8	3.5 – 5.7
Aspartate transaminase (AST)(SGOT) (U/L)	30	13 – 39
Alanine Transaminase (ALT) (U/L)	43	7 – 52
A/G Ratio	1.55	0.76 – 1.76
Total bilirubin (mg/dL)	0.3	0.0 – 1.0
Lipase (U/L)	722	11 – 82
LIPID PANEL		
Total cholesterol (mg/dL)	578	< 200
Triglycerides (mg/dL)	>3,000	< 150
HDL cholesterol (mg/dL)	12	> 40
CHOL HDL-C ratio	48.2	≤ 5
VLDL (mg/dL)		5 – 30
Non-HDL cholesterol (mg/dL)	566	< 130
LDL cholesterol (mg/dL)	175	0 - 129

**Figure 1:**
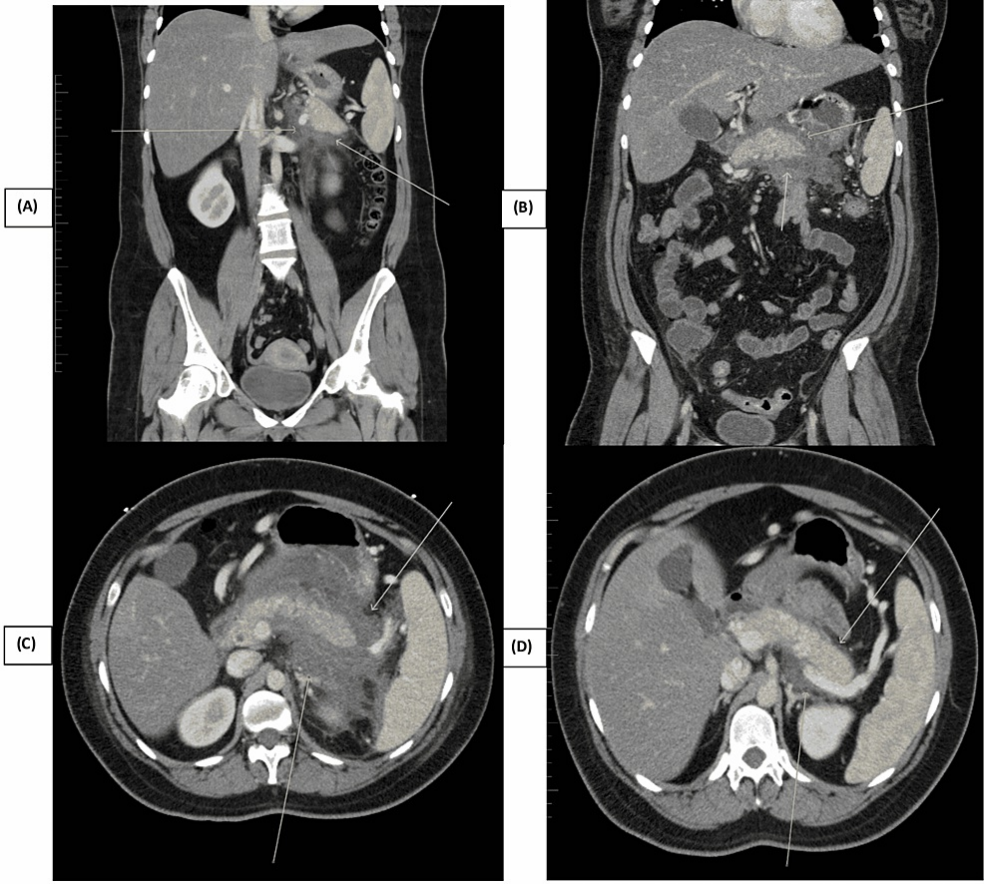
CT abdomen and pelvis showing acute pancreatitis with hepatic steatosis (A-D)

She had elevated serum lipase, i.e., 722 U/L (normal: 11-82 U/L) and amylase levels, i.e., 161 U/L (normal: 29-103 U/L), suggesting acute pancreatitis. Computed tomography (CT) of the abdomen and pelvis with contrast confirmed acute pancreatitis with hepatic steatosis (Figures 1A-1D). Ultrasound of the gallbladder was performed to rule out gallstone-induced pancreatitis; it showed a small amount of sludge in the gallbladder and a fatty liver. A general surgeon was consulted to evaluate the biliary sludge in the gallbladder. The patient was admitted for acute pancreatitis with a high suspicion of hypertriglyceridemia-induced acute pancreatitis. Other possible causes of acute pancreatitis, including alcohol consumption, drugs, viral infection, toxins, autoimmune pancreatitis, abdominal trauma, pancreatic divisum, and sphincter of Oddi dysfunction (SOD), were ruled out, thereby making hypertriglyceridemia-induced acute pancreatitis the most likely diagnosis.

She was placed nil per os (NPO), and intravenous (IV) fluids with normal saline were administered. Ranson's criteria for pancreatitis at the time of presentation showed two points with a predicted mortality of 1% (Table 2). The patient was initially admitted to the medical-surgical floor and subsequently developed systemic inflammatory response syndrome (SIRS). She was transferred to the intensive care unit (ICU) for close monitoring. She was started on IV insulin with 5% dextrose in normal saline. Serial basic metabolic panel (BMP) and triglyceride monitoring were done. A hydromorphone hydrochloride patient-controlled analgesia (PCA) pump was used for pain control. 

**Table 2:** Ranson criteria WBC: white blood cells; LDH: lactate dehydrogenase; AST: aspartate transaminase; SGOT: serum glutamic-oxaloacetic transaminase; BUN: blood urea nitrogen; PaO_2_: partial pressure of oxygen [5]

Admission Criteria	Criteria at 48 hours after admission
Age > 55 years	Hematocrit drop > 10%
WBC > 16,000/mm^3^	BUN rise > 5 mg/dL
LDH > 350 IU/L	Calcium < 8 mg/dL
Glucose > 200 mg/dL	PaO_2_ < 60 mmHg
AST (SGOT) > 250 U/L	Fluid sequestration > 6 liters

**Figure 2:**
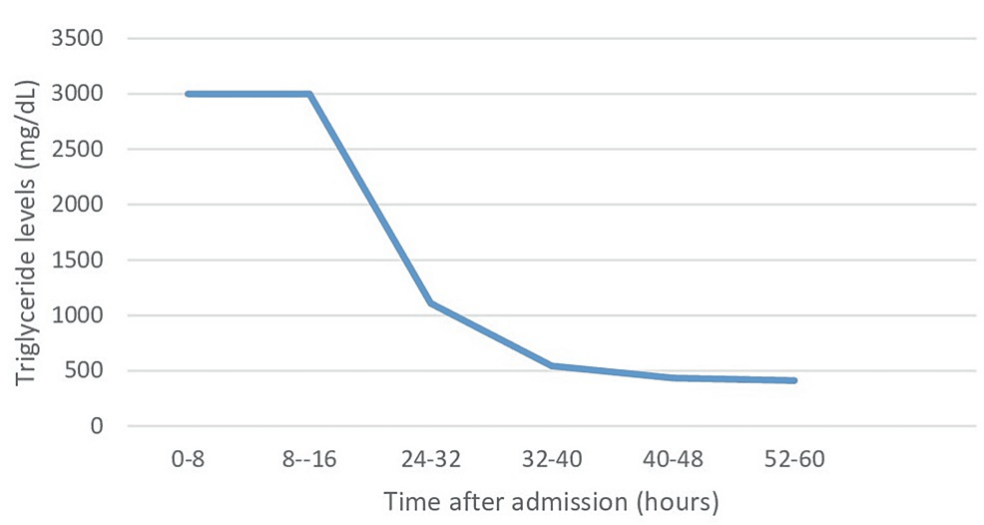
Changes in serum triglyceride levels over time mg/dL: Milligrams per deciliter

Ranson's criteria at 48 hours showed four points with a 15% predicted mortality. The patient's condition improved gradually. Her symptoms of abdominal pain and nausea resolved. Her triglyceride levels dropped to 1108 mg/dL within 24 hours of starting IV insulin and to 434 mg/dL in the next 24 hours (Figure 2). LDH levels 24 hours later were 238 mg/dL (normal: 140-271 U/L). Calcium was replaced as needed during the hospitalization. Pain medications were slowly tapered and a clear liquid diet was introduced and advanced as tolerated on the fifth day of hospitalization. At the time of discharge, the triglyceride (311 mg/dL) and lipase levels (81 U/L) of the patient were within the normal range. AST levels also normalized to 22 U/L by the time of discharge.

## Discussion

While acute pancreatitis has many etiologies, hypertriglyceridemia is known to be the third most frequent cause after gallstones and alcohol abuse. The diagnosis of acute pancreatitis requires at least two of the following features: (a) acute epigastric pain radiating to the back, (b) rise in pancreatic enzymes two-to-three times above the normal value, and (c) acutely inflamed pancreas on imaging. Hypertriglyceridemia-induced acute pancreatitis poses a diagnostic challenge when triglyceride levels are below 1,000 mg/dL as they are found to be mildly elevated in all cases of acute pancreatitis, regardless of the underlying cause [6]. Several cases of acute pancreatitis induced by moderate hypertriglyceridemia (<1,000 mg/dL) have been reported [1,7]. However, significantly elevated triglyceride levels (>1,000 mg/dL) should prompt physicians to consider hypertriglyceridemia-induced acute pancreatitis, particularly when other possible etiologies have been ruled out. Our patient had findings on imaging consistent with acute pancreatitis, a family history of hypertriglyceridemia, markedly elevated serum triglyceride levels, as well as no alcohol consumption, gallstones, new medication, ERCP, or abdominal trauma. Therefore, hypertriglyceridemia was diagnosed on the basis of these findings.

Although the clinical presentation of hypertriglyceridemia-induced acute pancreatitis is similar to that of other etiologies, the prognosis is suggested to be poor compared with other causes [4,6]. In a meta-analysis conducted by Wang et al., hypertriglyceridemia-induced acute pancreatitis was significantly associated with higher mortality (OR=1.90; 95% CI: 1.05, 3.45; p<0.01) and higher rates of SIRS (OR=2.03; 95% CI: 1.49, 2.75; p<0.00001) compared with non- hypertriglyceridemia-induced acute pancreatitis [4]. Our case is in concordance with this finding as our patient also developed SIRS and required ICU admission.

The exact pathophysiology of hypertriglyceridemia-induced acute pancreatitis remains to be elucidated. The most widely accepted mechanism involves the release of free fatty acids by the hydrolysis of excess triglycerides by pancreatic lipases. As these free fatty acids overwhelm the binding capacity of albumins, they cause damage to pancreatic acinar cells, platelets, and vascular endothelial cells. The acidic environment produced by subsequent ischemia further exacerbates the free fatty acid toxicity. Another implicated mechanism is increased blood viscosity due to hypertriglyceridemia which impairs pancreatic blood flow, leading to further ischemia and pancreatic damage. Moreover, hypertriglyceridemia may also result in acute pancreatitis by worsening the endoplasmic reticulum stress [6].

It is essential to timely and effectively manage patients with hypertriglyceridemia-induced acute pancreatitis in order to avoid complications [1]. One of the commonly used treatments is insulin infusion especially if plasmapheresis is unavailable or not tolerated by the patient. Insulin is effective in reducing triglyceride levels by increasing the activity of lipoprotein lipase and inhibiting hormone-sensitive lipase in adipocytes [3]. Our patient was already on chronic treatment with fenofibrate as she was a known case of hypertriglyceridemia. IV administration of insulin resulted in a diminution of her symptoms and a reduction in triglyceride levels. The patient was on the waiting list for a higher center for plasmapheresis. The patient recovered on Insulin drip before a bed was available at a higher center.

## Conclusions

Patients with hypertriglyceridemia-induced acute pancreatitis need swift diagnosis and treatment to avoid serious complications. The current case highlights the effective use of intravenous insulin with dextrose in an emergency setting in a patient with recurrent acute pancreatitis who was a known case of hypertriglyceridemia. This calls for further investigations to elucidate the efficacy of insulin in the treatment of hypertriglyceridemia-induced acute pancreatitis. Recurrence of acute pancreatitis should be prevented by lifestyle modification and appropriate lipid-lowering therapies.
